# Letter to the Editor: Total parenteral nutrition-induced Wernicke’s encephalopathy after oncologic gastrointestinal surgery

**DOI:** 10.1515/med-2024-0920

**Published:** 2024-02-28

**Authors:** Oguzhan Koca, Zeynep Hande Turna

**Affiliations:** Department of Internal Medicine, Cerrahpasa Medical Faculty, Istanbul University-Cerrahpasa, Kocamustafapasa Street No:53 Fatih, 34098 Istanbul, Turkey; Division of Medical Oncology, Department of Internal Medicine, Cerrahpasa Medical Faculty, Istanbul University-Cerrahpasa, Istanbul, Turkey

**Keywords:** WE, parenteral nutrition, cancer, thiamine replacement, oncologic gastrointestinal surgery

**To the editor,**

We read the article “Total parenteral nutrition-induced Wernicke’s encephalopathy after oncologic gastrointestinal surgery” with great interest. In this article, Fedeli et al. present a table with patients who developed Wernicke’s encephalopathy (WE) after oncologic gastrointestinal surgery.

In the case report by Onieva-González et al. [1], the patient underwent cephalic pancreaticoduodenectomy, and WE occurred postoperatively. However, the reason for pancreaticoduodenectomy was not pancreatic cancer but a duodenal ulcer penetrating the pancreas causing massive upper gastrointestinal bleeding. Moreover, the authors did not report histological data. Similarly, Iwase et al. [2] showed that thiamine levels were lower in patients who underwent gastrectomy; nonetheless, none of the patients presented WE. Therefore, these reports may not be included in the table.

Few cases of WE after oncologic gastrointestinal surgery have been reported in the literature. In turn, many cases of WE after non-oncologic gastrointestinal surgery (e.g., bariatric surgery) have been described. Notwithstanding the type of patients (alcoholic vs non-alcoholic or oncologic or non-oncologic), WE is associated with impaired thiamine uptake in the gastrointestinal tract.

We performed a brief literature review using the PubMed interface. We searched cases of WE or Wernicke–Korsakoff syndrome in patients with a history of oncologic gastrointestinal surgery. We included all the cases regardless of publication date. In addition to the cases in this article, 35 other case reports of WE after oncologic gastrointestinal surgery were found in the literature [3,4,5,6,7,8,9,10]. A total number of 51 cases of WE after oncologic gastrointestinal surgery were examined. Cancer types of these cases were gastric cancer, pancreatic cancer, colorectal cancer, gall-bladder cancer, duodenal cancer, ampulla of Vater cancer, and cholangiocellular cancer. Surgery techniques were subtotal gastrectomy, total gastrectomy, pancreaticoduodenectomy, anterior resection, abdominal-perineal resection, gastro-entero-anastomosis, hemicolectomy, cholecystectomy, subtotal stomach-preserving pancreatoduodenectomy, Roux-en-Y hepaticojejunostomy, and gastrojejunostomy. Most of these cases have not had the classic triad of confusion, ocular abnormality, and ataxia. Permanent neurological deficits occurred in cases where thiamine replacement was delayed [10,11]. Therefore, patients with malignancies, especially those who have undergone gastrointestinal surgery, are at increased risk of thiamine deficiency. In cancer patients with encephalopathy/confusion, WE should be suspected in addition to common etiologies such as infections, metastasis, and cerebrovascular events.

WE is not uncommon in cancer patients, especially those who have undergone gastrointestinal surgery. The European Federation of Neurological Societies guideline has reported that 18.1% of non-alcoholic WE is caused by cancer [12]. However, recommendations on prophylactic thiamine replacement in cancer patients who underwent gastrointestinal surgery are lacking. The National Comprehensive Cancer Network guidelines (version 1.2023) for gastric cancer make recommendations for vitamin B12 and iron replacement but not for thiamine replacement [13]. The recommendation for the treatment of WE is 500 mg thiamine administration three times per day until symptoms resolve [11], and the recommendation for the prevention of WE in high-risk patients is 100 mg thiamine administration three times per day [14].

In conclusion, thiamine deficiency and WE are common in cancer patients who underwent gastrointestinal surgery, and treatment delay can lead to permanent neurological deficits. Moreover, recommendations on thiamine replacement should be included in clinical practice guidelines in oncology. Furthermore, studies are needed to determine the effectiveness of preventive strategies in high-risk patients.
